# Trigeminal Neuralgia: Frequency of Occurrence in Different Nerve Branches

**DOI:** 10.5812/kowsar.22287523.2164

**Published:** 2011-09-26

**Authors:** Tanweer Hussain Bangash

**Affiliations:** 1 Employee Health Department, Peshawar, Khyber Pakhtoonkhwa, Pakistan

**Keywords:** Trigeminal neuralgia, Neuropathic pain, Neuralgia

## Abstract

**Background:**

Trigeminal neuralgia (TN) is neuropathic pain which can involve any part or side of the face.

**Objectives:**

The objectives of this study were to find the most common branch of trigeminal nerve affected and the most common side involved.

**Patients and Methods:**

This Cross sectional study was carried out on 100 patients of trigeminal neuralgia in one year time. The diagnosis was based on a detailed history, clinical examination and control of pain by carbamazepine being taken supplemented by radiographic investigations. The collected data was analysed by SPSS 16.

**Results:**

The age of the patients varied from 40 to 80 years with a mean age 54 years at the time of presentation. The males to females ratio was 1:2. The right side of the face was found to be involved in seventy patients (64 %) and left side in (36 %). No case presented with bilateral involvement. The Mandibular division was most commonly involved in this study (n = 55; 55%) and least was ophthalmic divisions (n = 6; 6%).

**Conclusions:**

This study demonstrated numerous clinical similarities of trigeminal neuralgia afflicting different populations. Right side was more involved along with mandibular division the most commonly affected. However studies needs to be done to know the exact reasons of involvement of the affected side and branches.

## 1. Background

Trigeminal neuralgia (TN) is characterized by a short-lasting, sharp electric-shock-type pain that arises from one or more branches of the trigeminal nerve (1-6). Most cases are of the primary or idiopathic type. Intracranial lesions that cause compression or traction of the trigeminal nerve are uncommon, but are a recognized cause of secondary trigeminal neuralgia. The incidence of trigeminal neuralgia is about 4. 5 per 100,000 people per annum, the ages of peak incidence being the 60s and 70s (Table 1) (2, 4, 5, 7). Attacks of TN are usually initiated by mild mucocutaneous stimulation in the territory of the affected trigeminal nerve, called the trigger zone (8). Most patients respond well to carbamazepine. Second-line pharmacotherapies include baclofen, gabapentin, lamotrigine, and phenytoin. For medically intractable TN, microvascular decompression is generally the treatment of choice, but radiosurgery and other ablative techniques are also used and are successful in relieving neuralgia in the majority of patients. Right-sided facial affliction predominates with the mandibular nerve being most commonly involved (5, 7, 9-11).

**Table 1.** Age and Gender Distribution in Trigeminal Neuralgia

Age, y	Male	Female	Total
40–50	9	19	28
51–60	10	17	27
61–70	12	21	33
71–80	3	9	12
Total	34	66	100

## 2. Objectives

The aim of the study was to determine the most common branch of the trigeminal nerve involved, and the sidedness, in TN.

## 3. Patients and Methods

This cross-sectional study was carried out on 100 patients with TN who presented at the department of Oral and Maxillofacial Surgery, Khyber College of Dentistry, Peshawar, from December 2008 to December 2009. All patients provided informed consent after the procedure to be performed was explained verbally. The diagnosis was based on a detailed history, clinical examination, and on the use carbamazepine for pain control. An orthopentograph (OPG) was performed for every patient to exclude any pathology. The branch of nerve was identified according to the site of pain, and confirmed with a diagnostic injection of local anesthetic (2% lignocaine with adrenaline, 1:200,000) at the identified site, repeated 3 times on consecutive days. An early morning appointment was given to all patients. The collected data was analyzed by the SPSS 16 software package (Cary, NC, USA).

## 4. Results

**Figure 1.**
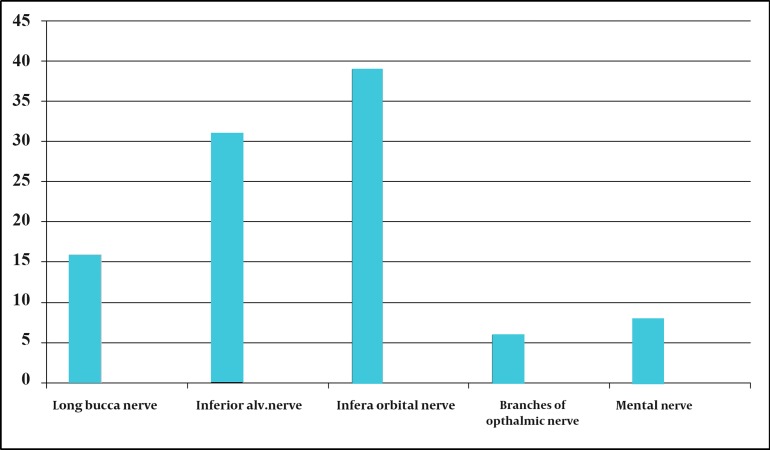
Distribution of Nerve Branches

The age of the patients varied from 40 to 80 years with a mean of 54 years at the time of presentation. The ratio of males to females was 1:1. 9. The right side of the face was found to be involved in 64 patients (64%) and the left side in 36 patients (36%). No case presented with bilateral involvement. The mandibular division was most commonly involved (n = 55; 55%), followed by the maxillary (n = 39; 39%) and ophthalmic divisions (n = 6; 6%). The combination of V2 and V3 was seen in only 9 patients. The combined involvement of all 3 divisions was not seen in our study. The numbers of cases in which the involvement of each of the nerve branches was seen were as follows: mental, n = 8; inferior alveolar, n = 31; long buccal, n = 16; infra-orbital, n = 39 (Figure 1). 

## 5. Discussion

TN is a condition likely to increase in prevalence, and to continue to challenge general practitioners and geriatricians alike. TN has an incidence of 4–5 per 100,000 of the population. It is nearly twice as common in women, and the incidence increases with age to around 1 in 1000 patients older than 75 years (12). Upon literature review, it is interesting to note that 3 reports from India demonstrated a male predominance (13-15). Katusic (16) reported female predominance in the ratio of 5. 9:3. 4. Other reviewers have reported similar findings (5, 9, 16, 17). The present study showed that the ratio of males to females suffering from TN was 1:2, which is consistent with the results of Loh et al. (18) and Shah et al. (8) All these previous studies have reported that the peak age of onset is between the fifth and eighth decades of life (12, 13, 16, 17, 19, 20). This trend was also seen in the current study, with the observed peak age being between the sixth and seventh decades of life. This concurrence supports the cardinal rule of subjecting patients under 40 years of age who complain of neuralgia-like pain in the face to a detailed neurological assessment to exclude associated diseases like multiple sclerosis (12, 16, 17, 19, 20). The age range at presentation in this study was 40 years to 80 years. The mean age was found to be 54 years. There is some controversy about the frequencies of presentation in the right and left sides. The right side of the face is more commonly affected than the left (ratio of 1. 5:1), possibly because of the narrower foramen rotundum and foramen ovale on the right side (7, 9, 10). We also found pain presentation to be higher on the right side (64%) than on the left side (36%). Bilateral presentation was not seen in our study. These results were surprisingly the same as those from a previous study by Shah et al. (8). Most of the published studies have revealed that the mandibular division (V3) was most commonly involved and that the ophthalmic division (V1) was less commonly presented (5, 7, 9-12, 15-24). Further, all support the typical feature of this condition in which the mandibular and maxillary (V2) divisions are more commonly involved than the ophthalmic. Shankland et al. (23) reported that a third of the patients in their study presented with neuralgic pain involving both the second and third divisions of the fifth nerve. Some of the patients in the present study also had both the mandibular and maxillary divisions being simultaneously affected.

The frequency of involvement of the maxillary division remained in between those of the other two. Casey et al. (21) found that symptoms were predominant in the V3 (15%), V2 (17%), and the combination of V3 and V2 (32%), and rarely started in the V1 alone. All 3 divisions were affected in 17% of patients at onset. In this investigation, the following findings were reported. Only 6% of patients presented with involvement of the first division, whereas the third division was the most common (55%). The combined involvement of V2 and V3 were seen in only 9 patients. All the 3 divisions were not involved in combination in our study. This was consistent with the study conducted by Shah et al. (8) However Katusic et al. (16) showed the involvement of the mandibular and maxillary divisions of the trigeminal nerve in approximately equal proportions, and in a small percentage of the ophthalmic division. No specific reason could be attributed to higher or lower involvement of nerve branches in the disease. This study demonstrated numerous clinical similarities of TN afflicting different populations. Careful history and identification of the nerve involved is important for accurate diagnosis and is essential to satisfactory treatment. Regular follow-ups should be carried out for TN patients, so as to change the treatment plan if needed.
